# Emerging Players at the Intersection of Chondrocyte Loss of Maturational Arrest, Oxidative Stress, Senescence and Low-Grade Inflammation in Osteoarthritis

**DOI:** 10.1155/2018/3075293

**Published:** 2018-02-11

**Authors:** Manuela Minguzzi, Silvia Cetrullo, Stefania D'Adamo, Ylenia Silvestri, Flavio Flamigni, Rosa Maria Borzì

**Affiliations:** ^1^Dipartimento di Scienze Mediche e Chirurgiche, Università di Bologna, Bologna, Italy; ^2^Laboratorio di Immunoreumatologia e Rigenerazione Tissutale, Istituto Ortopedico Rizzoli, Bologna, Italy; ^3^Dipartimento di Scienze Biomediche e Neuromotorie, Università di Bologna, Bologna, Italy

## Abstract

The prevalence of Osteoarthritis (OA) is increasing because of the progressive aging and unhealthy lifestyle. These risk factors trigger OA by removing constraints that keep the tightly regulated low turnover of the extracellular matrix (ECM) of articular cartilage, the correct chondrocyte phenotype, and the functionality of major homeostatic mechanisms, such as mitophagy, that allows for the clearance of dysfunctional mitochondria, preventing increased production of reactive oxygen species, oxidative stress, and senescence. After OA onset, the presence of ECM degradation products is perceived as a “danger” signal by the chondrocytes and the synovial macrophages that release alarmins with autocrine/paracrine effects on the same cells. Alarmins trigger innate immunity in the joint, with important systemic crosstalks that explain the beneficial effects of dietary interventions and improved lifestyle. Alarmins also boost low-grade inflammation: the release of inflammatory molecules and chemokines sustained by continuous triggering of NF-*κ*B within an altered cellular setting that allows its higher transcriptional activity. Chemokines exert pleiotropic functions in OA, including the recruitment of inflammatory cells and the induction of ECM remodeling. Some chemokines have been successfully targeted to attenuate structural damage or pain in OA animal models. This represents a promising strategy for the future management of human OA.

## 1. Introduction

Osteoarthritis (OA) is the most frequent arthritic disease with an expanding prevalence not only due to the aging of the population in western countries and to the increasing impact of some key epidemiological factors such as the global epidemic of obesity and metabolic syndrome (and the consequent increase of the so-called metabolic syndrome-associated OA or metOA) but also to increased exposure to independent risk factors that are associated to the postindustrial era [1].

OA heavily impacts on the expenditure of the national health services as well as on patients' quality of life. Currently, there are not yet true disease-modifying drugs and in most cases the disease progresses until arthroplasty.

The bone-joint decade (2000–2010) has fostered the research aimed at a better understanding of musculoskeletal disorders there including OA in order to improve prevention and treatment. Therefore, the recent years witnessed the deepening of our knowledge of the molecular mechanisms that are responsible for this disease. It becomes clear that notwithstanding the initial OA triggering risk factors (aging, mechanical stress, genetics, and obesity), there are shared downstream molecular modifications that affect transcription factors and epigenetic changes, thus leading to common dysregulation of target gene expression in joint cells and disrupted cellular functions [2]. Moreover, it is now a matter of fact that OA represents a disease of the whole joint organ, with important crosstalks between various tissues. There is also an increasing awareness of the “systemic” dimension of OA, which is supported by the evidence of crosstalks between “local” joint tissue inflammation and “systemic” inflammation. This is in the frame of a generalized failure of postmitotic tissues supported by shared mechanisms, such as the impairment of major homeostatic cell responses [3, 4] that lead to accumulation of oxidative stress, cell dysfunction, and senescence. These stress signals are perceived as “danger-associated molecular pattern” and thus are able to trigger macrophage activation and innate immunity. Notably, a recent proteomic study performed in the sera of OA patients pointed at a cluster of three proteins as highly indicative of radiographic OA and therefore useful as convenient biomarkers for screening and early diagnosis. This cluster contains an alarmin (S100A6) and a complement protein (C3), two proteins able to trigger innate immunity, besides inter-alpha-trypsin inhibitor heavy chain 1 (ITH1) that acts as a protease inhibitor [5].

A putative successful therapeutic strategy should therefore combine several lines of actions targeted at multiple levels with the aim of restoring “joint” homeostasis in the context of a “systemic” homeostasis. This implies dramatic changes in lifestyle (diet, exercise, healthy microbiota, and weight loss) that ultimately have the potential of reducing the activation of the innate immunity in joint and nonjoint tissues.

Basic research deepened our understanding of OA pathogenesis, and major achievements in defining critical targets have been obtained in matching findings derived from human tissues with those derived from several different animal models. A wide array of OA animal models either surgical or chemical have been developed, but the destabilization of medial meniscus (DMM) carried out in mice at the time of skeletal maturity is recognized as the method of choice for mimicking human pathology and its progression from mild-to-moderate OA (4 weeks) to moderate-to-severe OA (8 weeks) [6]. DMM should be a first choice to challenge mice with gene deletions of potential targets in OA [7].

Many review papers have reported an updated understanding of the role of signaling pathways [8] or cytokines [9] in OA pathogenesis, but a cumulative picture of cellular and molecular mechanisms of oxidative stress in articular cartilage in relation to aging, activation of innate immunity, metabolism, and cellular survival/longevity was lacking. In this review, we summarized recent information linking aging, metabolic deregulation, oxidative stress, and inflammation, with particular focus on selected chemokines, which have a role in pain. As long as OA can be considered as resulting from loss of tissue homeostasis, the above-mentioned pathogenetic mechanisms all result in an accelerated cartilage aging.

## 2. OA in the Context of Aging and Metabolic Deregulation

### 2.1. OA and Loss of Maturational Arrest

OA is the joint disease most closely correlated with aging. The status of healthy articular cartilage is actively kept in virtue of molecular constraints that prevent the progression of chondrocytes into their default route to hypertrophy and terminal differentiation prior to endochondral ossification [10]. Indeed, along OA initiation and progression, chondrocytes undergo a switch in gene expression from an “anabolic” to a “catabolic” pattern [10], recapitulating what occurs in growth plate calcification, the latter being considered as a “developmental model” for OA pathogenesis [11]. Chondrocyte progression from a “resting” to an “activated” state in either growth plate calcification or OA cartilage degeneration is indeed accompanied by the same shared signature of marker genes (extracellular matrix (ECM) proteins or transcription factors) and related phenotypic changes and cell reaction patterns such as cell proliferation or apoptotic cell death [12]. The loss of this “maturational arrest” of articular cartilage in all the species is somehow programmed to occur at a given age that follows the decline of the reproductive age and depends on the life expectancy of the species. On a general basis, life expectancy and longevity of a species have been connected to the differential capacity of its cells to handle oxidative damage to the DNA [13–16] in the form of double-strand breaks.

### 2.2. Oxidative Stress, NF-*κ*B Activation in OA, and Differential IKK Role

The role played by oxidative damage is central in OA and in the other age-related diseases. Indeed, at the cell level, oxidative damage of genomic and mitochondrial DNA (mtDNA) triggers a DNA damage response and activation of the nuclear factor-*κ*B (NF-*κ*B) pathway [3, 17], the master regulator of inflammation. NF-*κ*B is a family of transcription factors that have a central role not only in the proinflammatory stress-related responses of chondrocytes to extra- and intracellular insults but also in the control of their differentiation program [17, 18]. The activation of NF-*κ*B is triggered by the signalosome complex, a molecular switch controlled by two main serine threonine kinases able to remove NF-*κ*B inhibitors that block NF-*κ*B transcriptional activities: IKK*β* and IKK*α*. These kinases present peculiar functional features, with regard to the type of upstream activating stimuli, signaling intermediates, and downstream NF-*κ*B activated heterodimers. IKK*β* is the kinase that triggers the “canonical pathway” of NF-*κ*B activation that controls most of the immediate inflammatory, stress-like responses *in vivo* and *in vitro*. IKK*α* is required for the noncanonical or alternative pathway, which is delayed and requires proteolytic processing. In some cellular settings, IKK*α* may also intervene in the canonical inflammatory pathway [19]. Moreover, as reviewed in [17, 20], IKK*α* has other functions that boost NF-*κ*B transcriptional activities, including the inhibition of histone deacetylases and the phosphorylation of serine 10 on histone H3, thereby promoting a transcriptionally permissive conformation of the chromatin. H3 phosphorylation is required to activate promoters of some inflammatory genes, there including IL-6, IL-8, and MCP-1 [21]. These activities are played by IKK*α* in the nucleus, and indeed, quoting from http://proteinatlas.org, IKK*α* “is mainly localized to the nucleoplasm and in addition localized to the cytosol and vesicles” (https://www.proteinatlas.org/ENSG00000213341-CHUK/cell), while IKK*β* is only detected in the cytosol (https://www.proteinatlas.org/ENSG00000104365-IKBKB/cell) in keeping with its activity almost only implicated in the framework of NF-*κ*B activation. The pleiotropic activities of IKK*α* are presented in http://reactome.org and span far beyond signal transduction, differently from IKK*β*.

Our work has also recently highlighted other IKK*α* activities relevant for OA that are kinase and NF-*κ*B independent and that control ECM remodeling [22, 23] via collagenase activation. Therefore, IKK*α* targeting could be effective in reducing OA progression, yet sparing the prosurvival and antiapoptotic activities that are instead uniquely controlled by IKK*β* [20]. Indeed, pharmacological IKK*α* inhibition attenuated OA progression in the DMM model, where OA progression itself is accompanied with increased expression of IKK*α* [24].

IKK*α* is therefore a candidate drug target for human OA. IKK*α* could be locally targeted to obtain at the same time reduction of p53 [25] and NF-*κ*B activity [17, 20], together with the inhibition of its ECM remodeling promoting effects [23].

### 2.3. The IKK*α*/p53 Connection, Oxidative DNA Damage, Obesity, and Cell Senescence

**Figure 1:**
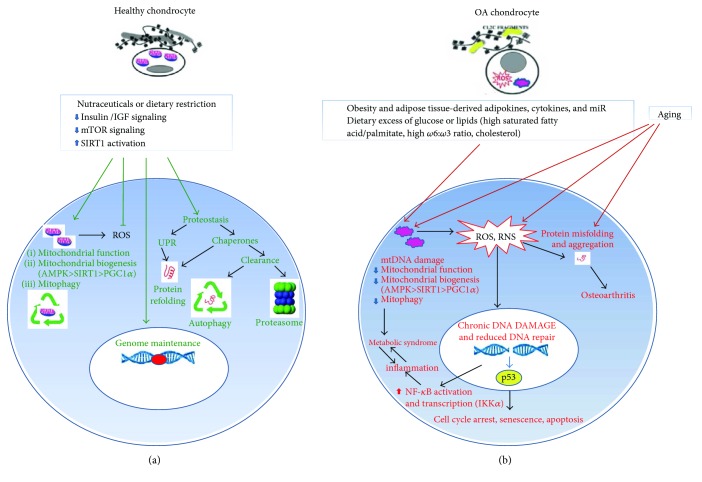
Failure of major homeostatic mechanisms contributes to OA pathogenesis. Failure of major homeostatic mechanisms (proteostasis and DNA damage repair networks and mitochondrial respiratory metabolism) in articular chondrocytes is caused by aging or conditions that accelerate tissue aging, such as the obesity-associated systemic low-grade inflammation and/or dietary factors (dyslipidemia and/or hyperglycemia) that impact on mitochondrial function. (a) Homeostasis is tightly controlled by balance between the insulin/IGF_mTOR signaling and SIRT1. In conditions of homeostasis normal mitochondrial function, biogenesis and autophagy (mitophagy) guarantee that the level of ROS is kept to the minimum required for intracellular signaling. Both genomic and mtDNA are preserved from oxidative damage. Proteostasis is guaranteed by the correct functioning of the unfolded protein response and clearance via autophagy or the ubiquitin-proteasome system. (b) Deranged metabolic factors together with aging contribute to mitochondrial dysfunction, accumulation of ROS and RNS that increase the level of protein misfolding and aggregation, and impact on the integrity of both mitochondrial and genomic DNA. Accumulation of DNA damage cannot be efficiently corrected because mitochondrial dysfunction leads to failure of the energy supply required by the DNA damage response. Persistent DNA damage is responsible for chronic NF-*κ*B activation, and inflammation, leading to the “metabolic syndrome.” A positive feedback loop between metabolic syndrome and inflammation is even worsened by excessive ROS and RNS produced by the dysfunctional mitochondria. Persistent DNA damage is also responsible for p53 activation, with functional consequences for the cells that include cell cycle arrest, senescence, or apoptosis according to an increasing degree.

The connection linking oxidative stress and inflammation has been reviewed in [26]. This results in cell dysfunction at multiple levels [3]. The DNA damage response activates and stabilizes p53 through posttranslational modifications and prevents its degradation by proteasome. An intriguing crosstalk has been described between IKK*α* and p53: under exposure to oxidative stress, the redox-sensitive PKC*δ* activates IKK*α* and induces its translocation to the nucleus, where it phosphorylates and activates p53 [25]. As a function of the DNA damage magnitude, p53 may orchestrate homeostatic activities or dysfunctional responses, such as irreversible cell cycle arrest, senescence, or apoptosis [27] (Figure 1). A recent report explored the correlation of gene and protein expression of cyclin D1, cyclin-dependent kinase 4, and p53 in cartilage derived from a large cohort of patients and healthy controls and showed that cyclin D1 and cyclin-dependent kinase 4 are negatively correlated with the disease grade in knee OA, in agreement with increased p53 [28].

Oxidative damage of genomic DNA and of mtDNA has been found in OA cartilage [29, 30]. Noteworthy, the level of oxidative damage to mtDNA has been found correlated with BMI of the patients and associated to markers of DNA damage response, hypertrophy, and senescence [29]. This discloses a molecular pathogenesis peculiar for the so-called metOA whose development is indeed anticipated compared to posttraumatic, aging, genetic, or crystal OA phenotypes [31]. Metabolic syndrome (MetS) is a global epidemic (23% of the general population). White adipose tissue (WAT) plays an endocrine role in MetS: increased M1 macrophages and adipose-derived stem cells (ASC) release inflammatory cytokines, chemokines, and adipokines. Adipocytes and possibly other WAT cells [32] release circulating microRNAs (miR). Circulating (blood or exosomal) miR dysregulation in MetS has been already reported [33]. Among their putative targets, there are proteins which may afford protection in conditions of metabolic or oxidative stress, such as AMP-activated protein kinase (AMPK), PPAR*γ*, and PKC*ε* [33]. Their dysfunction might result in increased DNA damage and senescence in ASC, conditioning their stemness and their trophic activity while dysfunction of these proteins in postmitotic tissues such as cartilage disrupts the existing cellular homeostasis [34, 35]. Reduced AMPK*α*1 [36] and PPAR*γ*1 [37] expression in chondrocytes as a function of OA severity has been already reported. Moreover, adipose tissue has a role in OA development, acting as an endocrine organ and producing adipokines and inflammatory mediators. Leptin activates the JAK/STAT pathway and proinflammatory response and chondrocytes express functional leptin receptors [38]. Many studies evidenced a role for leptin in altering chondrocyte function, via increased expression of ECM degradative enzymes, namely, matrix metalloproteinases and enzymes of the “a disintegrin and metalloproteinase with thrombospondin motifs (ADAMTS)” family. Leptin has been pointed out as a necessary factor for OA development in *in vivo* animal models of obesity, while studies on patients support its role in human OA, with a direct correlation between serum leptin levels and diminished cartilage thickness [39, 40].

### 2.4. Metabolic Deregulation in OA Pathogenesis and the Nutraceutical Approach to OA Management

Obesity is associated with a status of systemic inflammation affecting the function of many postmitotic tissues, there including the cardiovascular system. Indeed, the incidence of OA is doubled in people with cardiometabolic clustering (higher or equal to 2 of the following: low levels of high-density lipoprotein cholesterol; elevated levels of low-density lipoprotein cholesterol, triglycerides, blood pressure, C-reactive protein, waist-to-hip ratio, or glucose; or diabetes mellitus) [41]. This opens new perspectives in the treatment of these diseases that share similar mechanisms leading to target tissue cell dysfunction and boosts the relevance of developing therapeutic approaches in the perspective of a precision medicine. On a general basis, the use of mere antioxidant supplements in clinical trials has led to weak and inconsistent results [42], while an integrated lifestyle approach including dietary intervention with nutraceutical implementation, physical exercise, and caloric restriction may increase cell homeostatic activities [43].

The ability of some nutraceuticals (in particular polyphenols) to affect chondrocyte homeostatic activities includes functional effects at multiple levels in the cells, interfering with major inflammatory signaling pathways [44] and exerting a nutrigenomic/epigenetic control [45]. Recent evidence has shown the ability of hydroxytyrosol to influence the epigenetic regulation of small noncoding RNAs, thus rescuing homeostatic activities in chondrocytes [46, 47].

Many deranged metabolic factors such as an hyperlipidemic environment, high levels of cholesterol and glucose, and oxidized low-density lipoproteins may directly impact on chondrocyte health and contribute to OA pathogenesis [48].

High dietary intake of total fat and saturated fatty acid (SFA) has been recently put in correlation with progression of knee OA [49]. The same study also reported that dietary intervention with higher intake of monounsaturated fatty acid (MUFA) or polyunsaturated fatty acid (PUFA) or higher PUFA/SFA ratio was associated with reduced OA progression and joint space narrowing. Another critical factor is represented by the plasma *ω*6 : *ω*3 ratio that positively correlates with pain and inflammation [50]. In obesity-associated OA, adipose tissue functions as an endocrine organ and contributes to low-grade inflammation, also enhanced by a M2 to M1 switch of resident macrophages. Importantly, *ω*6 or *ω*3 PUFA entering the same metabolic route in adipocytes yields proinflammatory or anti-inflammatory mediators, respectively [51], with relevant systemic consequences and differential impacts on joint tissues. The mechanisms underlying these effects on OA patients have been explored *in vitro* and *in vivo* in animal models.

**Figure 2:**
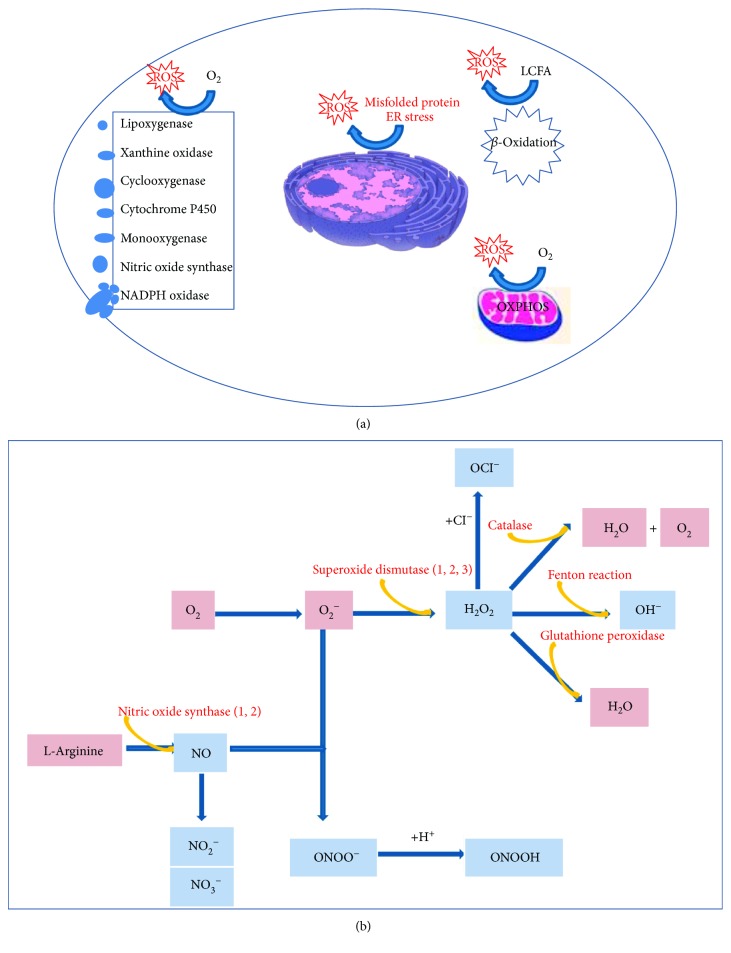
(a) Sources of ROS in the chondrocyte. The major sources of ROS are the mitochondria, during the OXPHOS. In addition, ROS can be generated at the level of the endoplasmic reticulum (ER), in condition of ER stress. An additional source is represented by the peroxisome, during the *β*-oxidation of long chain-fatty acids (LCFA), particularly under conditions of excess LCFA load. Various enzymes, with different cell location may also generate ROS. (b) Production of ROS and RNS involved in OA pathogenesis, and major detoxifying enzymes. The first generated ROS is the superoxide (O_2_^−^) that is either rapidly transformed into hydrogen peroxide (H_2_O_2_) by the enzymes of the superoxide dismutase (SOD) family or can act as a precursor for peroxynitrite (ONOO^−^) formation when joined to nitric oxide (NO), produced by the enzymes of the nitric oxide synthase (NOS) family. Because of unpaired electrons, free radicals are very reactive and can damage cell components. In chondrocytes, iron Fe^2+^ and H_2_O_2_ release hydroxyl radicals (OH^−^) that react with unsaturated fatty acids of membrane lipids, thus forming lipid radicals (RO^•^, ROO^•^). ROS are neutralized by scavenging systems, namely, SOD, catalase, glutathione peroxidase, glutathione reductase, and reduced glutathione.

The dose-dependent increased release of reactive oxygen and nitrogen species (ROS and RNS) and inflammatory cytokines by chondrocytes exposed to mixtures of free fatty acids (FFA) including palmitate has been recently shown *in vitro* [52] and OA development in rats fed with high-SFA diet has been reported *in vivo* [53] (see Figure 2). Palmitate has also proapoptotic and proinflammatory activities [54] that are reduced after inhibition of Toll-like receptor (TLR) 4.

Recent evidence has also indicated that high cholesterol levels impact on mitochondrial health, thus leading to increased ROS production and an anabolic-to-catabolic switch. This condition is amenable for treatment with mitochondria-targeted antioxidants [55]. Moreover, a higher expression of miR33a and its host gene SREBP2 in OA compared to normal cartilage has been reported, together with information of the contribution of this axis to OA pathogenesis [56, 57].

The hyperglucidic-mediated dysregulation of chondrocytes has also been recently connected to derangement of the Nrf-2/HO-1 axis, a fundamental pathway in antioxidant protection [58]. Moreover, sustained hyperglycemia leads to increased level of glucose derivatives (advanced glycation end-products (AGEs), sorbitol, and diacylglycerol (DAG)) able to promote the activation of several pathways triggering inflammatory processes as reviewed in [59].

High levels of fatty acids or glucose may also worsen their damaging activity by additional mechanisms, there including a depressive activity of key metabolic stress regulators. In adipose tissue, AMPK is rescued by *ω*3 PUFA [51]. High levels of glucose may contribute to an altered metabolism in OA chondrocytes favoring glycolysis and depressing oxidative phosphorylation. This leads to impaired mitochondrial metabolism and drives to mitochondrial damage, associated to decreased levels of AMPK, SIRT-1, and PGC-1*α*, the master regulator of mitochondrial biogenesis [60] (Figure 1). PPAR*γ* is also reduced by high levels of glucose in chondrocytes [61].

### 2.5. Proteostasis and Metabolic Stress Management

Aging is also characterized by a collapse in proteostasis, intended as “protein homeostasis,” a tight regulation of protein fate from biogenesis to degradation. Proteostasis is guaranteed by the correct functioning of the unfolded protein response (UPR), autophagy, the ubiquitin-proteasome system, and the lyosomes. Alteration in these pathways is a source of stress for proteins, endoplasmic reticulum (ER), and mitochondria (Figure 1). Proteostasis impairment is involved in OA pathophysiology [62], since it leads to increased oxidation of proteins and alteration of homeostatic pathways.

Chaperone and cochaperone proteins intervene in maintaining the “proteostatic network,” altered in aging and disease. Several stressors are able to trigger the heat shock response pathway (HSR) [63]. Its activation implicates chromatin remodeling to promote the transcription of heat shock proteins (HSPs), thus increasing stress resistance. HSPs are a family of highly conserved proteins, classified according to their molecular weight (HSP100, HSP90, HSP70, HSP60, HSP40, and small HSPs). They orchestrate proteome maintenance not only in de novo folding and protein trafficking but also in protein degradation and in preserving autophagic machinery components [64]. Failure of the proteostasis network is a programmed event that occurs in both development and in aging [65]. Indeed, altered chaperone expression has been observed in aged animal models and in senescent human cells [63]. A significant decrease in HSP70 expression has been reported in senescent cells derived from degenerated human intervertebral disc annulus [66].

AMPK and SIRT1 are the main bioenergy sensors that not only react to changes in energy balance but also in inflammatory processes and cell stress. SIRT1 is reduced by aging, and this impacts on proteostasis via reduction of the activity of the heat shock factor 1 (HSF1) and of the FOXO pathway [67]. Impairment of AMPK activity in cartilage promotes ECM degradation. AMPK has been found reduced in human OA as a consequence of low-grade inflammation [36]. AMPK is also affected by age and age-related accumulation of DNA breaks. The latter indeed induce DNA PK phosphorylation of HSP90*α* that is then able to exert an AMPK-inhibiting activity [68].

Specific increase and activation of HSP factors like HSP70 and HSF1 have been observed in synovial tissues of rheumatoid arthritis but not in OA [69]. However, in a rat OA model, increased expression of HSP70 as induced by the proteasome inhibitor MG132 reveals a local protective effect on matrix degradation and apoptosis [70]. This is in keeping with evidence pointing at a chondroprotective role of the chaperone protein HSP70 against biomechanically induced OA in rats [71]. The protective activity of HSP60 is instead acted through the reduction of SOX9 ubiquitination [72]). TRAP1, a chaperone protein of the HSP90 family with antioxidant and antiapoptotic activities and specifically expressed in mitochondria, was found upregulated in human OA chondrocytes [73]. On the other hand two proteomic studies of another group have instead identified two small HSPs that are reduced in OA chondrocytes to an extent that is associated with the severity of the disease. *α*B-cristallin-reduced expression impacts on ECM homeostasis [74], while the small chaperone HSP27 is involved in chondrocyte response to IL-1*β* [75].

SIRT1 expression is reduced in both human OA cartilage and in knee cartilage of both OA mouse model and aged mice, thus strongly suggesting SIRT1 involvement in OA development. Indeed, Sirt1 knockout mice developed accelerated OA progression both in cartilage-specific or whole organism genetic models [76–78]. Recently, a wide study on human OA cartilage has negatively correlated SIRT1 expression with disease severity [79]. Together with the pivotal role in maintenance of mitochondrial homeostasis [80], SIRT1 is involved in activating HSF1 transcription and proteostasis, particularly preventing ER stress. In OA, articular chondrocyte UPR defective activity enhances ER stress thus leading to misfolding of ECM proteins [62].

## 3. OA and Oxidative Stress and Senescence

### 3.1. Sources of ROS and Major ROS Scavenging Systems in the Cell

A recent definition of oxidative stress is “an imbalance between oxidants and antioxidants in favour of the oxidants, leading to disruption of redox signaling and control and/or molecular damage” [81] and is strongly associated with aging and age-related diseases, including OA [42].

ROS production occurs mainly in mitochondria through the mitochondrial respiratory chain and the oxidative phosphorylation (OXPHOS), but ROS can also be generated by NADPH oxidase, xanthine oxidase, and other sources (Figure 2(a)). It has been shown that the expression of some of these prooxidant enzymes increases in synovial membrane of OA patients [82]. The NADPH oxidase family comprises different members with peculiar cell localization, ROS products, and regulation. Among others, a function of the NADPH enzymes is to act as key modulators of signal transduction pathways. One emerging activity is the reversible posttranslational oxidative modification of several proteins such as via sulphonylation of conserved cysteine residues. This however may shift into a pathological role after excessive activation under chronic stress. Indeed, NADPH oxidase 4 (Nox4) has been recognized as contributing to OA pathogenesis, since its overexpression leads to increased ROS and matrix metalloproteinases (MMPs) [83] and has been proposed as a therapeutic target in OA.

The first generated ROS is the superoxide (O_2_^−^) (Figure 2(b)) that is either rapidly transformed into hydrogen peroxide (H_2_O_2_) by the enzymes of the superoxide dismutase (SOD) family [84] or acts as a precursor for peroxynitrite formation (ONOO^−^) when joined to nitric oxide (NO), produced by the enzymes of the nitric oxide synthase (NOS) family [85].

The main cartilage damaging ROS detected in chondrocytes are hydrogen peroxide and peroxynitrite [86, 87]. Because of unpaired electrons, free radicals are very reactive and can damage cell components. In chondrocytes, Fe^2+^ and H_2_O_2_ release hydroxyl radicals (OH^•^) that react with unsaturated fatty acids of membrane lipids, thus forming lipid radicals (RO^•^, ROO^•^). ROS are neutralized by scavenging systems, namely, SOD, catalase, glutathione peroxidase, glutathione reductase, and reduced glutathione (Figure 2(b)). Several studies carried out in human OA cartilage and experimental animal models reported a limited antioxidant capacity of SOD, catalase, and the glutathione systems [88, 89]. Therefore, oxidative stress leads to membrane and nucleic acid damage and, also, breakdown of extracellular components, including proteoglycans and collagens. In addition, oxidative stress further sustains an anabolic-to-catabolic switch, with downregulation of glycosaminoglycan and collagen synthesis and upregulation of MMP and aggrecanase release. The final outcome is cartilage derangement and destruction. The evidence of mitochondrial dysregulation is supported by a characteristic mitochondrial protein profile in proteomic analysis of human OA samples. This confirms that redox imbalance is a key factor in OA pathogenesis, as evidenced by a reduction in SOD2 (the mitochondrial SOD) expression in superficial cartilage area [73].

### 3.2. Oxidative Damage to Genomic and Mitochondrial DNA and Cell Senescence

The increased ROS burden of aged cells [90] may be responsible for oxidative damage of both genomic and mtDNA. However, it is recognized that mtDNA damage is more extensive and persists longer than nuclear DNA damage in human cells [91, 92]. The key role of oxidative damage to mtDNA in OA is supported by the evidence of differential disease susceptibility of different mtDNA haplogroups [93, 94]. In addition, mitochondrial biogenesis is impaired in OA [95]. Therefore, chronic oxidative stress maintains a positive feedback loop with mitochondrial dysfunction, increased ROS, and persistent mtDNA damage, recognized to be a hallmark of chronic degenerative diseases. This emphasizes the critical role of autophagy of dysfunctional mitochondria (mitophagy) in the rescue of chondrocyte homeostasis. The mtDNA damage is in turn responsible for the senescence of OA chondrocytes, a condition of proliferative arrest not due to telomere shortening after a limited number of division (intrinsic senescence) but rather to an accumulation of DNA damage (extrinsic senescence) following exposure to stress-inducing factors.

Occurrence of *in vivo* telomere shortening in OA chondrocytes has been reported by some investigators [96]. Indeed, since the ends of chromosomes are particularly sensitive to oxidative stress, this can result in telomere shortening similar to that seen with replicative senescence [97, 98]. Recently, rather than to mature chondrocytes, critical susceptibility to telomere attrition and senescence has been attributed to a pool of progenitor cells whose magnitude increases along disease progression [99]. Telomere attrition in OA chondrocytes has been questioned by other studies that instead found that increased oxidative or genotoxic stress leads to “stochastic” genomic DNA damage in the OA compared to normal chondrocytes, responsible for heterogeneity of gene expression in chondrocytes with no evidence of critical telomere shortening [30].

The effects of oxidative stress have been evaluated *in vitro* by other studies. Susceptibility to the oxidative stimuli depends on culture conditions. A fundamental issue to keep in mind is that chondrocytes change their differentiation status *in vitro*; therefore, to obtain reliable information, it is important to grow them in culture conditions that allow to keep/recover a proper phenotype, that is, at high density or in 3-D [100]. It is interesting to underline that chondrocytes are inherently quite resistant to oxidative stimuli (e.g., H_2_O_2_) that induce apoptosis in a dose- and time-dependent manner [101]. To obtain significantly increased chondrocyte apoptosis at high density, as much as 500 *μ*M H_2_O_2_ must be kept for 24 hours [101], or in any case, a consistent time window must be allowed to observe the effect.

Telomere attrition *in vitro* has also been evaluated: chondrocytes cultured within their native ECM were exposed to a long-lasting oxidative stimulus. Then, the cells were recovered from the tissue by means of enzymatic digestion, and H_2_O_2_-induced telomere attrition was assessed [102] together with the protective effect of ascorbate delivery.

Senescent OA chondrocytes express the senescence-associate secretory phenotype (SASP) [103] that features an overproduction of inflammatory cytokines, matrix degrading enzymes, and ROS and is sustained by the upregulation of the inflammatory pathways that participate in the “aging stress response” [3]. Therefore, senescent chondrocytes are able to affect the surrounding microenvironment, determine a senescent status of cartilage precursor cells, and even trigger OA when transplanted in healthy joints [104]. This suggests that targeting senescent cells could be an attractive therapeutic strategy in OA. Indeed, a recent report elegantly demonstrated that in a model of surgically induced OA, a significant number of senescent cells accumulate in cartilage and synovium, and selective targeting of these cells with the newly described senolytic drugs reduces progression, pain, and inflammatory/degradative activities, while promoting a regenerative environment [105].

Furthermore, along with differentiation progression and under the effects of inflammatory stimuli, human articular cartilage shows increased expression of senescence markers [106] that correlates with disease severity [107]. In keeping with this information, epigenetic (miR-mediated) mechanisms link senescence and chondrocyte terminal differentiation-associated matrix remodelling in OA [108]. Noteworthy, a healthier phenotype may be rescued through p16^INK4a^-specific siRNA *in vitro* [106].

An increase of oxidative activity of the mitochondria is also sustained by inflammatory stimuli that lead to glycogen synthase kinase 3 (GSK3) inhibition [109]. Indeed, inactivation of GSK3 is responsible for mitochondrial activation, persistent production of mitochondrial ROS through decreased complex IV activity, and cell senescence [29, 110]. Inflammation and oxidative stress are interdependent and boost each other, sustained by ROS production and antioxidant depletion [26]. In line with this concept, expression of the NALP3 inflammasome has been found strictly correlated with that of prooxidant enzymes in the synovium of knee OA [82], thus further confirming that oxidative stress sustains inflammation and degradation of the articular cartilage.

## 4. Low-Grade Inflammation in OA

### 4.1. The Role of Innate Immunity and of the Macrophages in OA

Only a few decades ago, the disease that represents the leading cause of chronic disability of the elderly was called “osteoarthrosis,” to indicate the prevalence of degenerative over inflammatory processes in its pathogenesis, at least with regard to lack of the classical signs *rubor et tumor cum calor et dolor* that are instead evident in the so-called inflammatory arthritides. However, an expanding body of findings collected in recent years points at the molecular evidence of inflammation in OA chondrocytes. These cells have been resembled to activated macrophages because of the shared inflammatory mediators that are able to release, and also other functional properties [111].

In recent years, increasing evidence of the pivotal role of NF-*κ*B [17, 22] in OA pathogenesis has shed light on several molecular mechanisms responsible for cartilage derangement. Interestingly, enhanced and coordinated *in vivo* expression of the NF-*κ*B target gene inducible nitric oxide synthase (iNOS) and the prototypical inflammatory cytokines (IL-1*β* and TNF*α*) in OA chondrocytes or in AR synoviocytes indicated that the major focus of OA inflammation was the cartilage, as opposed to the synovium in inflammatory arthritides [112]. However, to date, OA is considered as a whole organ disease of the joint, with important crosstalks from the synovium and the subchondral bone [113]. Occurrence of synovitis with mononuclear cell infiltration always precedes the development of radiological changes in human OA while in animal models of surgically induced OA, ablation of factors critical for macrophage recruitment ameliorates OA progression [114], thus hinting at a role for “innate” immunity in OA pathogenesis. Indeed, in both human (patients with meniscal tears, a condition that may precede OA development) and animal DMM [6] models of OA initiation, the extent of synovitis is correlated with pain and dysfunction and its molecular characterization reveals a unique chemokine signature [115, 116]. The chronic and low-grade inflammation present in OA synovium is primarily innate and secondarily adaptive. Despite the extent of the infiltrate being lower in OA compared to RA, some features are characteristic of OA synovium: the number of mast cells is higher [117]. Alteration of both conventional and nonconventional Th subsets suggests a role of T cells in OA onset and progression [118]. A recent paper indicated that in infrapatellar fat pad there is an increase of infiltrating CD8+ T cells that correlates with the radiological score [119]. B cells, plasma cells, natural killer cells, dendritic cells, and granulocytes are also occasionally present. Innate immunity is the first line of defense in the body that intervenes when physical and chemical barriers fail. Innate immunity is mostly driven by the recruitment and activation of macrophages that trigger inflammation with the aim to get rid of the *noxae*. However, a targeted macrophage depletion strategy proved to be unsuccessful and even worsened the inflammation status suggesting that macrophages also exert homeostatic activities [120].

### 4.2. Alarmins and Chemokines

Macrophages detect conserved pathogen motifs (PAMPs or pathogen-associated molecular patterns) via invariable pattern recognition receptors (PRRs). The best known class of PRRs corresponds to special receptors known as Toll-like receptors (TLRs), able to recognize bacterial or viral components such as lipopolysaccharide (LPS) or double-stranded RNA (dsRNA). TLR activation then induces macrophages to secrete cytokines (small molecules involved in cell signaling and attraction), as well as to phagocytose the infected cells. The innate immune system is also required to activate the “adaptive” immune system. TLR activation involvement in both synovitis activation and OA development has been extensively reviewed in [121, 122] and connected to ECM degradation products such as hyaluronan and fibronectin.

PRRs are also able to recognize damage-associated molecular patterns (DAMPs or alarmins), molecules that are produced and released during tissue damage with the aim of orchestrating tissue repair. DAMPs include the high-mobility group box 1 (HMGB1) and the S100 family proteins. Their pivotal role in disease development has been recently elucidated [123].

Alarmins are able to trigger both synovial macrophages (via TLR2 and TLR4) and chondrocytes (via TLR2, TLR4, and RAGE), thus sustaining both cartilage degradation and synovial inflammation. The crucial role of alarmins is supported by the evidence that the development of collagenase-induced OA has been blocked by the delivery of an inhibitor of S100A9 [124].

Among alarmins, HMGB1 has been shown to increase chemokine production by chondrocytes [125]. Moreover, HMGB1 has the ability to form complexes with either IL-1 or LPS, thus enhancing their inflammatory effects on synovial fibroblasts [126]. HMGB1 is a highly conserved nonhistonic nuclear protein that primarily acts as a chromatin binding factor to promote protein assembly at specific DNA targets. HMGB1 has important intracellular and extracellular functions. HMGB1 is passively released from necrotic cells and actively secreted by activated macrophages. In cartilage, stage-specific HMGB1 expression and release regulate endochondral ossification [127]. At the extracellular level, HMGB1 may serve as an inflammation promoting cytokine but also as a danger signal, and its potential as a therapeutic target in rheumatic diseases has already been reported [128–130]. In comparative studies of mRNA expression of normal versus OA cartilage, an inverse trend was found between the procatabolic HMGB1 and the proanabolic HMGB2. Besides cartilage, HMGB1 is expressed also by synovial cells, and its concentration in the synovial fluid has been related to OA severity [131].

**Figure 3:**
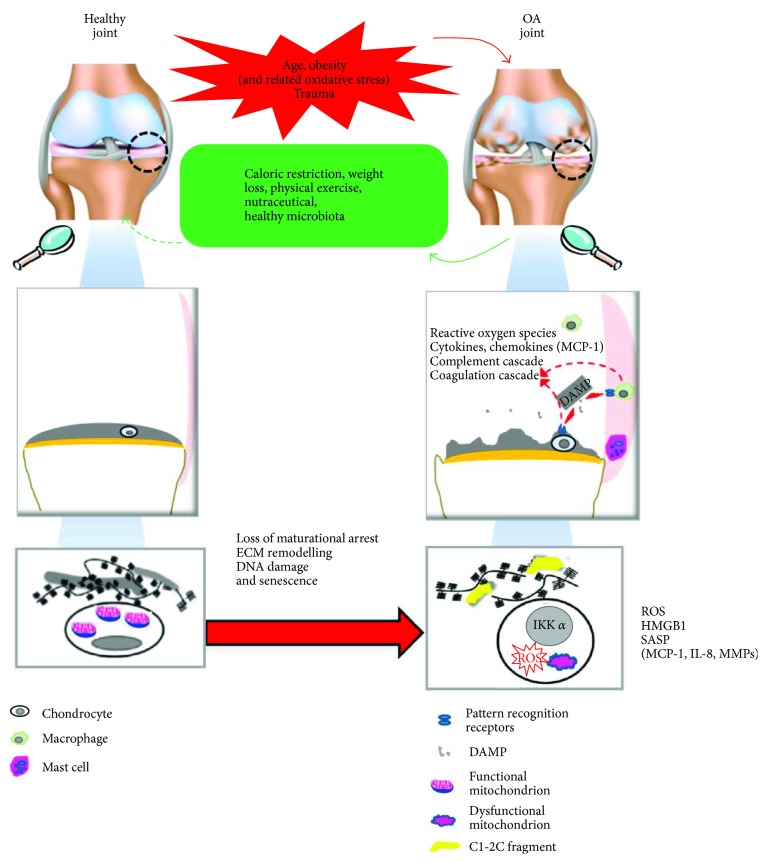
Macro to micro views of changes occurring in articular cartilage during OA onset and development. Left images indicate healthy joint, joint tissues, and chondrocytes. Resting chondrocytes present functional mitochondria and are surrounded by intact ECM. As a consequence of different risk factors, profound changes occur in all the joint tissues. Right images indicate OA joint, crosstalk among OA joint tissues, and hypertrophic chondrocytes. The latter present dysfunctional mitochondria, increased ROS production, and increased IKK*α* expression and are surrounded by degraded ECM. ECM degradation products act as damage associated molecular patterns (DAMPs or alarmins) recognized by the pattern recognition receptors (PRR) expressed by the cells. Alarmins are able to trigger both synovial macrophages (via TLR2 and TLR4) and chondrocytes (via TLR2 and TLR4, and RAGE), thus sustaining synovial inflammation and cartilage degradation. DAMPs also include HMGB1 that is highly expressed by both OA synoviocytes and hypertrophic chondrocytes and is able to enhance NF-*κ*B activation and release of chemokines (particularly of MCP-1, highly active in the recruitment of monocytes that are also present in the synovial fluid) and cytokines, as well as of matrix metalloproteinases (MMPs) responsible for ECM remodeling and loss of maturational arrest. Increased oxidative stress is both cause and consequence of mitochondrial dysfunction. Correction of major risk factors and changes in diet and lifestyle can significantly reduce disease progression.

Addition of HMGB1 to cultures of human chondrocytes dramatically increased the expression of certain known NF-*κ*B targets, including iNOS and the chemokines that are selectively increased in OA chondrocytes, that is, IL-8, MCP-1, MCP-2, MIP-3*α*, MIP-1*α*, RANTES, and GRO*α* [125]. Interestingly, most of these chemokines belongs to the SASP, namely, the subset of molecules that show high increase (more than 4-fold) [103] (Figure 3). The expression of most of these molecules appears increased in OA compared to normal chondrocytes [132] or under the effect of chondrocyte activation with inflammatory stimuli [133, 134]. Since hypertrophy plays a pivotal role in OA pathogenesis and hypertrophic chondrocytes release large amounts of HMGB1 [127], it is conceivable that this mechanism greatly contributes to maintain the deranged OA chondrocyte transcriptisome [135].

The expression of chemokine receptors on chondrocytes has been reported several years ago together with the first findings of an increased expression in OA compared to healthy cartilage of some of these receptors [136] and their contribution to OA pathophysiology [137–139].

Chemokines represent a large molecule family of increasing size and complexity [140] that have been firstly described as having a role in leukocyte traffic but now recognized as able to affect many functions in a variety of tissues, including joint tissues. Most of these molecules share some basic features: the small size, the ability to bind to seven transmembrane G-protein coupled receptors to activate intracellular responses and to exploit binding to sulfated glycosaminoglycans (abundant in cartilage ECM) that act as coreceptors and determine haptotactic gradients around the chondrocytes.

In healthy cartilage where ECM is intact and highly organized, chemokines (7–9 kDa) have a higher access to the embedded chondrocytes compared to larger size cytokines, such as IL-1*β* (17 kDa).

The role of chemokines in OA has been recently reviewed and disclosed on the basis of cumulative evidence derived from both findings obtained in patients and evidence obtained in animal models of OA [141]. The severity of human OA as assessed by mean of the radiological score has been found correlated with the synovial fluid level of selected chemokines: CCR2 ligands (CCL2/MCP-1); CCR5 ligands (CCL3/MIP-1*α*, CCL4/MIP-1*β*, and CCL5/RANTES); CCR7 ligands (CCL19/MIP-3*β* and CCL21/SLC), and CXCR4 ligand (CXCL12/SDF-1).

We will mainly focus on CCL2/MCP-1, as it is the major chemokine active in macrophage recruitment and also responsible for adipose tissue inflammation in obesity, where the size of adipocytes correlates with MCP-1 levels [51]. Serum and synovial CCL2/MCP-1 levels are also strictly correlated with the pain score [142]. The understanding of the molecular connections comes from studies carried out in DMM models that demonstrate upregulation of both CCL2/MCP-1 and CCR2 in the neurons of innervating dorsal root ganglia (DRG) in mice 8 weeks after DMM. The CCL2/MCP-1 production by DRG neurons is once again connected to molecular danger signals (S100A8 and *α*2 macroglobulin) [143] originated in the joint after DMM and that may diffuse to DRG and trigger TLR4 particularly on nociceptive neurons. CCL2/MCP-1 release from DRG is responsible for maximal macrophage infiltration at 8 weeks. CCR2 ablation indeed conferred protection with respect to pain but not with respect to cartilage degradation [143]. The same authors have recently shown that Ccl2 is the gene most strongly upregulated immediately after the DMM. Ccl2^−/−^ or Ccr2^−/−^ animals show an altered and delayed inflammatory response but similar chondropathy scores compared to their wild-type controls. Moreover, they show a significantly delayed occurrence of pain [144]. Collectively, these data suggest that the CCR2_ CCL2/MCP-1 axis may be targeted for improving OA patients' quality of life rather for controlling cartilage structural damage [144].

Other studies reported a different scenario, pointing at a pivotal role of the CCR2_CCL2/MCP-1 axis also in structural damage. Discrepancies of findings in DMM studies may be related to the experimental design, including susceptibility of the strain [7], details of gene knockout (either full or tissue-specific and either constitutive or inducible) or age at DMM surgery, and duration of OA development in association with the timing of monitoring (hours; 4, 8, 12, and 20 weeks). A recent study focused on the transcriptional reprogramming that follows DMM at early time, while vanishing at a later time. This study indicated that at two weeks a significant upregulation was found for the TGF signaling pathway and for complement and coagulation cascade genes [145]. The upregulation of TGF is of particular interest in the context of the activities of the CCL2/CCR2 axis. Indeed, in a rat model of posttraumatic OA, Appleton and collaborators found that TGF2 and CCL2/MCP-1 were both upregulated at 4 weeks, with the former inducing the expression of the latter and with pharmacologic inhibition of either TGF2 or CCL2/MCP-1 leading to attenuation of structural damage in both *in vivo* or *in vitro* studies [146]. Notably, while the complement cascade belongs to the family of the innate immunity effectors and has been recently attributed a relevant role in mediating OA damage [147], the coagulation cascade includes the activated protein C (APC), which may act as an activator of cartilage matrix metalloproteinases [148]. Another study carried out in the rat meniscal tear OA model confirmed a strong upregulation of CCL2/MCP-1 as early as day 3 [149].

The CCR2_CCL2/MCP-1 axis is critical for the recruitment of macrophages to the joint [135]. CCR2-positive cells (macrophages) are abundantly detected in human OA synovial tissues and invade and erode cartilage in mouse OA [135]. The CCR2_CCL2/MCP-1 axis is so strictly connected with the pathogenesis of OA that a differential susceptibility to this disease has been related to both disease-promoting and disease-protective SNPs [150].

Synovial tissue specimens derived from OA patients showed increased mRNA expression of many chemokines active on monocytes and increased protein expression of the corresponding chemokine receptors, but only CCL2/MCP-1 and not CCL5/RANTES is significantly elevated in synovial fluid of OA patients, suggesting that a hierarchical selectivity exists for monocyte recruitment in OA, as also confirmed in mouse OA pathogenesis [135].

Pharmacologic suppression of CCL2/MCP-1 expression or CCR2 signaling significantly reduces OA progression and relative cartilage structural damage via inhibition of monocyte recruitment to synovial tissue and macrophage accumulation. Interestingly, elevation of CCL2/MCP-1 in synovial fluid has been pointed at as a reliable biomarker for aseptic loosening of total knee arthroplasty and bone loss [151].

Disruption of the CXCR4_CXCL12/SDF-1 axis led to protection in a guinea pig model of spontaneous OA [152]. In this perspective, it is worthy to underline that circulating HMGB1 may strengthen the downstream effects of this axis, as previously reported [153].

An intriguing hypothesis is emerging that connects innate immunity with the pathogenesis of many chronic degenerative diseases including OA [154]. An unhealthy microbiota composition together with an altered gut permeability may allow for increased absorption and serum concentration of LPS. At the systemic level, LPS may activate TLR on macrophages and trigger complement activation yielding the production of bioactive components (C3a, C3b, and C5a) that further enhance macrophage activation. LPS is indeed found elevated in serum of OA patients and correlated to knee joint narrowing [155]. To date, this mechanism has been elegantly confirmed in rats in a study [156] that teased out the pathogenesis of metOA associated with high-fat diet and related alteration of microbiota composition. Indeed, the animals treated with high-fat diet had higher serum levels of LPS and fecal analysis pointed at the abundance of particular species (*Lactobacillus* and *Methanobrevibacter*) with strong predictive relationship with the modified Mankin score.

## 5. Conclusion and Perspective

Latest literature findings have greatly added to the picture of OA as a disease of accelerated aging of a postmitotic tissue with little or no reparative potential, but within a framework of systemic inflammation. Except for chronological issues, in a biological sense, aging can be controlled and attenuated with the adoption of dietary intervention and lifestyle measures that rescue the functionality of major homeostatic mechanisms. In this perspective, it is worthy to underline that state-of-the-art investigations revisited OA as belonging to the so-called mismatch diseases, whose prevalence has increased in the last decades because of higher exposure to conditions that are far from those that were present when our species has evolved. These conditions include circadian rhythms, lifestyle, physical exercise, and diet. These factors are amenable to correction, thus making OA much more preventable than currently considered [1].

In the lack of a true disease-modifying strategy, once OA is established, a complete joint *restitutio ad integrum* appears improbable, but much can be acted to slow OA progression and delay the time of arthroplasty as well as controlling pain and disability (as depicted in Figures 1 and 3).

Although targeting of selected cytokines or molecules proved to be effective in delaying structural damage, more promising are the strategies focused to counteract general mechanisms of cell aging, such as via the use of dietary restrictions or nutraceuticals able to reduce the magnitude of insulin/IGF and mTOR signaling in favour of a stronger sirtuin activation, thus exerting antiaging effects via rescue of mitochondrial functionality, genome maintenance, and proteostasis. A fundamental effect of this dietary strategy is to promote clearance of damaged mitochondria, via mitophagy [157, 158]. This at the same time allows reduction of oxidative damage and cell senescence [3, 46, 47]. As an additional strategy, correction of intestinal microbiota may be of help in maintaining the integrity of the intestinal barrier and reducing adsorption of LPS, thus smoothing innate immunity activation.
